# The TransFLUas influenza transmission study in acute healthcare - recruitment rates and protocol adherence in healthcare workers and inpatients

**DOI:** 10.1186/s12879-019-4057-5

**Published:** 2019-05-21

**Authors:** Hila Schwarz, Jürg Böni, Roger D. Kouyos, Teja Turk, Edouard Battegay, Malcolm Kohler, Rouven Müller, Heidi Petry, Hugo Sax, Rainer Weber, Allison McGeer, Alexandra Trkola, Stefan P. Kuster

**Affiliations:** 1 Division of Infectious Diseases and Hospital Epidemiology, University Hospital and University of Zurich, Raemistrasse 100 / HAL14 D6, 8091 Zürich, Switzerland; 2 Institute of Medical Virology, University of Zurich, Zurich, Switzerland; 3 Department of Internal Medicine, University Hospital and University of Zurich, Zurich, Switzerland; 4 Pulmonary Division, University Hospital and University of Zurich, Zurich, Switzerland; 5 Hematology, University Hospital and University of Zurich, Zurich, Switzerland; 6 University Hospital Zurich, Zurich, Switzerland; 7 Department of Microbiology, Sinai Health System, Toronto, Canada

**Keywords:** Influenza, Transmission, Asymptomatic infection, Surveillance, Healthcare-associated infection

## Abstract

**Background:**

Detailed knowledge about viral respiratory disease transmission dynamics within healthcare institutions is essential for effective infection control policy and practice. In the quest to study viral transmission pathways, we aimed to investigate recruitment rates and adherence of healthcare workers (HCWs) and hospital inpatients with a study protocol that involves prospective surveillance based on daily mid-turbinate nasal swabs and illness diaries.

**Methods:**

Single center prospective surveillance of patients and HCWs in three different hospital departments of a tertiary care center during an entire influenza season in Switzerland. Inpatients and acute care HCWs were asked to provide mid-turbinate nasal swabs and illness diaries on a daily basis. Study protocol adherence and recruitment rates were the primary outcomes of interest.

**Results:**

A total 251 participants (59 (23.5%) health care workers and 192 (76.5%) inpatients) were recruited from three different hospital wards. Recruitment rates differed between HCWs (62.1% of eligible HCWs) and inpatients (32.5%; *P* < 0.001), but not within HCWs (*P* = 0.185) or inpatients (*P* = 0.301) of the three departments. The total number of study-days was 7874; 2321 (29.5%) for inpatients and 5553 (70.5%) for HCWs. HCWs were followed for a median of 96 days (range, 71–96 days) and inpatients for 8 days (range, 3–77 days). HCWs provided swabs on 73% (range, 0–100%) of study days, and diaries on 77% (range 0–100%). Inpatients provided swabs and diaries for 83% (range, 0–100%) of days in hospital. In HCWs, increasing age, working in internal medicine and longer duration of total study participation were positively associated with the proportion of swabs and diaries collected. Adherence to the study protocol was significantly lower in physicians as compared to nurses for both swabs (*P* = 0.042) and diaries (*P* = 0.033). In inpatients, no association between demographic factors and adherence was detected.

**Conclusions** Prospective surveillance of respiratory viral disease was feasible in a cohort of inpatients and HCWs over an entire influenza season, both in terms of recruitment rates and adherence to a study protocol that included daily specimen collection and illness diaries.

**Trial registration:**

clinicaltrials.gov NCT02478905. Date of registration June 23, 2015.

## Background

Influenza remains the most common infectious disease cause of death in the developed world, causing up to 5′000 hospitalizations and 1′500 deaths in Switzerland annually [1]. Elderly persons, young children and persons with underlying medical conditions are at highest risk for adverse outcomes [2]. Despite barrier protection measures and vaccination campaigns among healthcare workers (HCWs), nosocomial acquisition of influenza is a well-known patient safety issue, and outbreaks of influenza are common in both acute and long-term care [3–8].

The epidemiology and transmission dynamics of influenza in hospitals, however, are poorly understood. In particular, it is not known how often asymptomatic or minimally symptomatic persons may transmit disease [9, 10]. This poses a problem because one-in-three influenza infections are thought to be asymptomatic [11]. If asymptomatic persons transmit influenza, vaccination of patients and HCWs before start of the influenza season, the permanent use of masks by HCWs during influenza season, and quarantine for previously exposed inpatients may be the only available measures to reduce the number of influenza transmission events in acute care hospitals [12–14]. Bridging this knowledge gap would be of major benefit to infection prevention and control recommendations, and may result in reduced morbidity and mortality associated with influenza in hospitals. Studying the question whether asymptomatic individuals transmit influenza virus in acute care, however, requires close monitoring of a tight group of HCWs and patients with a high participation rate and adherence to a study protocol that comprises close monitoring of disease activity, symptoms and contacts between individuals.

To define whether exposure to asymptomatic subjects with influenza infection constitutes a risk for influenza virus transmission in an acute care hospital setting, we designed the TransFLUas study, an active, prospective surveillance study of HCWs and inpatients. We collected mid-turbinate nasal swabs and influenza-like illness symptom diaries on a daily basis on dedicated study wards over an entire influenza season, with the goal to assess influenza transmission pathways in relation to influenza symptoms. In this report, we describe participant recruitment and adherence to the protocol during the 2015/2016 influenza season.

## Methods

### Study setting, design and procedures

We performed a single center prospective surveillance study of HCWs and inpatients in three different hospital units (pulmonology, hematology and internal medicine) at the University Hospital Zurich over the 2015/2016 influenza season. The University Hospital Zurich is a 900 beds university-affiliated tertiary care center that covers all specialties except orthopedic surgery and pediatrics. It serves a population of 400′000 inhabitants for primary and 1′443’000 for tertiary care [15].

We followed patients in these 3 units, as well as nursing staff (nurses and assistant nurses), corporate hospitality staff with direct patient contact and medical staff (attending physicians and those in training) working on the same wards during the influenza season. Influenza season was considered to start when Swiss national sentinel surveillance levels exceeded the national threshold for an influenza epidemic and ended when influenza levels fell below the epidemic threshold for two consecutive weeks. (https://www.bag.admin.ch/bag/de/home/krankheiten/ausbrueche-epidemien-pandemien/aktuelle-ausbrueche-epidemien/saisonale-grippe---lagebericht-schweiz.html). All HCWs and inpatients ≥18 years of age on the ward under surveillance were eligible for the study. HCWs were excluded if they planned to spend more than two consecutive weeks outside of Switzerland during the influenza season in order to secure consistency over time. HCWs were recruited by study personnel at staff meetings prior to the influenza season. Patients were approached by study personnel upon admission to the participating study ward. Patients who were not competent to consent were excluded. Participants were not compensated for study participation.

HCWs were asked to complete a baseline demographic questionnaire, asking about personal data (age, sex), household characteristics that may be associated with reduced (e.g. having received seasonal influenza vaccine, influenza vaccination history) or increased (e.g. household crowding index (defined as the number of people per household divided by the number of bedrooms), living with children in their household) risk of influenza, and underlying medical conditions (Charlson comorbidity index) and long-term medication [16]. A questionnaire was filled in for inpatients upon enrollment, covering baseline characteristics, including age, sex, date of admission, vaccination status and co-morbidities (Charlson comorbidity index). Length of hospital stay was calculated from discharge and admission dates after hospital discharge.

In consenting inpatients, study nurses collected mid-turbinate nasal swabs daily from the day of enrollment until discharge and filled in diaries covering signs and symptoms of influenza infection (including cough, sore throat, fever ≥38.0 °C, nasal congestion, weakness, headache, loss of appetite or myalgia) and contacts with other subjects with influenza symptoms. Patients were asked to self-collect swabs and fill in daily diaries for 2 days after discharge and send the items back to the study office. HCWs were asked to self-collect mid-turbinate nasal swabs and to fill in the illness diaries on a daily basis (including days off work) and drop the items in an inbox placed on each study ward. The self-collection of flocked mid-turbinate nasal swabs has been shown to be comparable to nasopharyngeal aspirates in children and adults [17–19]. Updates on the current status of the influenza epidemic and reminders to continue sending in swabs and diaries were sent to participating HCWs by email each week.

### Definitions

Recruitment rate was defined as the number of consenting individuals divided by the number of eligible HCWs and inpatients, respectively.

We defined adherence with the study protocol for each study participant as the number of swabs and illness diaries, respectively, that were submitted in relation to the number of samples/diary entries that the study participant was expected to submit based on the study protocol.

### Statistical analysis

Categorical data were tested for differences using Fisher exact tests, whereas continuous variables were tested using Wilcoxon rank sum tests or the Student’s t test, as appropriate. Multivariable linear regression analysis was used to determine predictors for adherence to the study protocol. Potential predictors among participant characteristics were considered for inclusion in multivariable models based on clinical judgment and previous hypotheses, with final models representing those that best balanced parsimony and fit. Data were analysed using Stata® version 13.1 (Stata Corporation, College Station, TX). Two-tailed *P*-values < 0.05 were considered statistically significant.

## Results

### Recruitment

In total, 251 participants, including 59 (23.5%) HCWs and 192 (76.5%) inpatients were enrolled in the study. Overall, 192 out of 591 (32.5%) eligible patients could be recruited, and 59/95 (62.1%) HCWs participated in the study (*P* < 0.001) (see Table 1). Recruitment rates did not differ between the three departments for either HCWs or inpatients.

**Table 1:** Recruitment rates of patients and healthcare workers enrolled in prospective surveillance of influenza infection, University Hospital Zurich, 2015/2016 influenza season

Participants	Department				
	All departments	Pulmonology	Hematology	Internal medicine	*P*-value
Patients	192/591 (32)	80/263 (30)	56/179 (31)	56/149 (38)	0.3
HCWs	59/95 (62)	19/33 (52)	17/33 (58)	22/29 (76)	0.18
Nurses	49	15	13	21	
Physicians^1^	9	3	4	1	
Other HCWs^2^	1	1	0	0	

Data are n (%). ^1^one HCW was working on all three wards. ^2^one HCW from corporate hospitality

Abbreviations: *HCWs* healthcare workers

### Healthcare worker and patient characteristics

HCWs and patient characteristics are shown in Tables 2 and 3, respectively. HCWs had a median age of 30.5 (range, 18.0–58.0) years and 50 (84.7%) were female. The median household crowding index was 1.3 (range, 0.5–3), and 11 (18.6%) had children < 18 years of age in their household. There were no significant differences in demographic or other characteristics between HCWs from the three different study wards.

**Table 2:** Healthcare worker characteristics on wards with active surveillance for influenza, University Hospital Zurich, 2015/16 influenza season

Characteristic	Department				
	All departments (*n* = 59)^1^	Pulmonology (*n* = 17)	Hematology (*n* = 19)	Internal medicine (*n* = 22)	*P*-value
Age, years, median (range)	30.5 (18.0–58.0)	30.5 (18.5–51.6)	31.1 (21.5–58.0)	26.7 (18.0–51.9)	0.52
Female sex	50 (84.7)	14 (82.4)	17 (89.5)	19 (86.4)	0.45
Profession					
Nurse	49 (83.1)	13 (76.5)	15 (79.0)	21 (95.5)	0.27
Physician	9 (15.3)	3 (17.7)	4 (21.1)	1 (4.6)	
Other	1 (1.7)	1 (5.9)	0 (0)	0 (0)	
Charlson comorbidity index, median (range)	0 (0–1)	0 (0–1)	0 (0–0)	0 (0–1)	0.88
Myocardial infarction	0 (0)	0 (0)	0 (0)	0 (0)	n.a.
Congestive cardiac insufficiency	0 (0)	0 (0)	0 (0)	0 (0)	n.a.
Peripheral vascular disease	0 (0)	0 (0)	0 (0)	0 (0)	n.a.
Chronic pulmonary disease	2 (3.4)	1 (5.9)	0 (0)	1 (4.6)	0.59
Conjuctive tissue disease	0 (0)	0 (0)	0 (0)	0 (0)	n.a.
Diabetes without complications	0 (0)	0 (0)	0 (0)	0 (0)	n.a.
Peptic ulcer disease	1 (1.7)	0 (0)	0 (0)	1 (4.6)	0.44
Chronic disease of the liver or cirrhosis	0 (0)	0 (0)	0 (0)	0 (0)	n.a.
Hemiplegia	0 (0)	0 (0)	0 (0)	0 (0)	n.a.
Moderate or severe kidney disease	0 (0)	0 (0)	0 (0)	0 (0)	n.a.
Diabetes with chronic complications	0 (0)	0 (0)	0 (0)	0 (0)	n.a.
Solid organ malignancy	0 (0)	0 (0)	0 (0)	0 (0)	n.a
Leukemia	0 (0)	0 (0)	0 (0)	0 (0)	n.a.
Lymphoma	0 (0)	0 (0)	0 (0)	0 (0)	n.a.
Moderate or severe liver disease	0 (0)	0 (0)	0 (0)	0 (0)	n.a.
Malignant tumor, metastasis	0 (0)	0 (0)	0 (0)	0 (0)	n.a.
AIDS	0 (0)	0 (0)	0 (0)	0 (0)	n.a.
Dementia	0 (0)	0 (0)	0 (0)	0 (0)	n.a.
Household crowding index, median (range)	1.3 (0.5–3)	1.3 (1–2.5)	1.3 (1–2)	1.3 (0.5–3)	0.99
Children (< 18 years) in household	11 (18.6)	5 (22.7)	1 (5.3)	5 (22.7)	0.24

Data are n (%), unless indicated otherwise. ^1^Sum of HCWs of different departments does not equal total number as one HCW worked on all wards and thus was not assigned to one single department

Abbreviations: *n.a.* not applicable, *AIDS* acquired immunodeficiency syndrome

**Table 3:** Patient characteristics

Characteristic	Department				
	All departments (*n* = 192)	Pulmonology (*n* = 80)	Hematology (*n* = 56)	Internal medicine (*n* = 56)	*P*-value
Age, years, median (range)	57.9 (18.3–94.1)	54.6 (18.3–79.5)	55.9 (21.0–87.6)	62.2 (29.4–94.2)	0.002
Female sex	80 (41.6)	32 (40)	25 (44.6)	23 (41)	0.86
Charlson comorbidity index, median (range)	2 (0–8)	1 (0–8)	2 (0–6)	2 (0–7)	0.15
Myocardial infarction	10 (5.2)	3 (3.7)	3 (5.4)	4 (7.1)	0.71
Congestive cardiac insufficiency	16 (8.3)	7 (8.7)	1 (1.8)	8 (14.2)	0.13
Peripheral vascular disease	12 (6.2)	2 (2.5)	1 (1.8)	9 (16)	0.006
Chronic pulmonary disease	78 (40.6)	64 (80)	5 (8.9)	9 (16)	< 0.001
Conjuctive tissue disease	0	0	0	0	n.a
Diabetes without complications	27 (14)	11 (13.7)	2 (3.6)	14 (25)	0.017
Peptic ulcer disease	2 (1)	1 (1.2)	0	1 (1.8)	0.50
Chronic disease of the liver or cirrhosis	3 (1.6)	0	0	3 (5.4)	0.07
Hemiplegia	2 (1)	1 (1.2)	0	1 (1.8)	0.67
Moderate or severe kidney disease	22 (11.5)	11 (13.7)	4 (7.1)	7 (12.5)	0.47
Diabetes with chronic complications	6 (3.1)	1 (1.2)	0	5 (8.9)	0.035
Solid organ malignancy	12 (6.2)	4 (5)	1 (1.8)	7 (12.5)	0.054
Leukemia	20 (10.4)	1 (1.2)	19 (33.9)	0	< 0.001
Lymphoma	7 (3.6)	1 (1.2)	5 (8.9)	1 (1.8)	0.10
Moderate or severe liver disease	16 (8.3)	7 (8.7)	4 (7.1)	5 (8.9)	0.81
Malignant tumor, metastasis	5 (2.6)	4 (5)	0	1 (1.8)	0.30
AIDS	2 (1)	0	1 (1.8)	1 (1.8)	0.49
Dementia	1 (0.5)	1 (1.2)	0	0	0.59

Data are n (%), unless indicated otherwise

Abbreviations: *n.a.* not applicable, *AIDS* acquired immunodeficiency syndrome

We enrolled 192 patients with a median age of 57.9 years (range, 18.3–94.1), and an overall median Charlson comorbidity index of 2 (range 0–8) (Table 3). Patients from the internal medicine ward were older than others and more likely to have peripheral vascular disease or diabetes, whereas patients with chronic pulmonary disease were more likely to be hospitalized on the pulmonology ward and patients with leukemia were almost exclusively admitted to the hematology ward.

### Adherence to the study protocol

Table 4 shows the adherence to the study measures in HCWs and inpatients. In total, 7874 study-days were recorded, 2321 (29.5%) for inpatients and 5553 (70.5%) for HCWs. The median number of study-days was 96 (range, 71–96) for HCWs and 8 (range, 3–77) for inpatients. The median number of HCW- or patient-days did not differ between the study wards.

**Table 4:** Adherence to study requirements (collection of swabs and diaries on a daily basis) of patients and healthcare worker enrolled in prospective surveillance of influenza infection, 2015/2016 influenza season

	Department				
	All departments	Pulmonology	Hematology	Internal Medicine	*P*-value
Days in study					
Total patients	2321	920	788	613	n.a.
Median days in study per patient, range	8 (3–77)	9 (3–77)	8 (3–65)	8 (3–34)	0.83
Total HCWs	5553	1587	1774	2096	n.a
Median days in study per HCW, range	96 (71–96)	96 (71–96)	96 (79–96)	96 (80–96)	0.45
Nurses	96 (93–96)	96 (94–96)	96 (93–96)	96 (96–96)	0.91
Physicians	82 (71–96)	82 (71–92)	85 (79–88)	80 (80–80)	0.79
Swabs					
*Patients*					
Total number of swabs in study	1469	592	471	406	n.a.
Median number of swabs per patient, range	5 (0–68)	4.5 (0–68)	5 (0–49)	5 (0–26)	0.84
Total number of swabs in hospital	1430	568	461	401	n.a.
Median number of swabs abs in hospital, range	4 (0–68)	4 (0–68)	5 (0–49)	5 (0–26)	0.73
Total number of swabs after discharge	39	24	10	5	n.a.
Median number of swabs after discharge, range	0 (0–4)	0 (0–4)	0 (0–3)	0 (0–2)	0.53
Swabs per day in study, %	60 (0–100)	60 (0–100)	60 (0–100)	60 (0–90)	0.86
Swabs per day in hospital, %	83 (0–100)	80 (0–100)	84 (0–100)	85 (0–100)	0.97
*HCWs*					
Total number of swabs in study	3513	787	1244	1390	n.a
Median number of swabs in study, range	69 (0–96)	42 (0–96)	69 (18–96)	77.5 (0–92)	0.29
Swabs per day in study, %	62 (0–100)	49 (0–100)	70 (20–100)	66 (0–96)	0.26
*Nurses*					
Total number of swabs in study	3150	694	1066	1390	n.a
Median number of swabs in study, range	75 (0–96)	65 (0–96)	71 (22–96)	78 (8–92)	0.56
Swabs per day in study, %	67 (0–100)	56 (0–100)	74 (23–100)	69 (8–96)	0.56
*Physicians*					
Total number of swabs in study	339	69	178	0	n.a
Median number of swabs in study, range	36 (0–92)	27 (0–42)	48.5 (18–63)	0	0.25
Swabs per day in study, %	43 (0–96)	26 (0–46)	53 (20–74)	0	0.25
Diaries					
*Patients*					
Total number of diaries in study	1470	593	471	406	n.a.
Median number of diaries in study, range	5 (0–68)	4.5 (0–68)	5 (0–49)	5 (0–26)	0.85
Total number of diaries in hospital, total	1431	569	461	401	n.a
Median number of diaries in hospital, range	4 (0–68)	4 (0–68)	(0–49)	5 (0–26)	0.75
Total number of diaries after discharge, total	39	24	10	5	n.a
Median number of diaries after discharge, range	0 (0–4)	0 (0–4)	0 (0–3)	0 (0–2)	53
Diaries per day in study, %	60 (0–100)	60 (0–100)	60 (0–100)	60 (0–90)	0.86
Diaries per day in hospital, %	83 (0–100)	80 (0–100)	84 (0–100)	85 (0–100)	0.97
*HCWs*					
Total number of diaries in study	3661	849	1275	1444	n.a.
Median number of diaries in study, range	72 (0–96)	49 (0–96)	72 (18–96)	83 (0–94)	0.30
Diaries per day in study, %	77 (0–100)	53 (0–100)	77 (20–100)	86 (0–98)	0.27
*Nurses*					
Total number of diaries in study	3275	740	1091	1444	n.a
Median number of diaries in study, range	78 (0–96)	69 (0–96)	75 (24–96)	83 (8–94)	0.59
Diaries per day in study, %	81 (0–100)	73 (0–100)	78 (25–100)	86 (8–98)	0.57
*Physicians*					
Total number of diaries in study	361	84	184	0	n.a
Median number of diaries in study, range	37 (0–93)	35 (0–49)	49 (18–68)	0	0.25
Diaries per day in study, %	47 (0–97)	43 (0–53)	60 (20–77)	0	0.25

Abbreviations: *n.a.* not applicable, *HCWs* healthcare workers

A total of 1469 mid-turbinate nasal swabs (median, 5; range, 0–68) were collected from participating inpatients, 1430 (97.3%) during hospitalization (median, 4; range, 0–68) and 39 (2.7%) after discharge. Twenty-seven (14.1%) inpatients provided swabs after hospital discharge. The median percentage of eligible swabs submitted per day in hospital was 83% (range, 0–100%) and did not differ between the three study wards. There was no association of adherence to the study protocol with age, sex, underlying comorbidities, ward or duration of participation in the study (data not shown).

HCWs self-collected a total of 3513 swabs (median, 69; range, 0–96). The median proportion of swabs per day in study among HCWs was 73% (range, 0–100%) and of diaries 77% (range 0–100%). Multivariable linear regression analysis revealed increasing age (*P* = 0.001), working in internal medicine as compared to pulmonology (*P* = 0.019) and longer duration of total study participation to be positively associated with the proportion of swabs collected. The same predictors were found for adherence with filling in study diaries (data not shown). Adherence to the study protocol was significantly lower in physicians as compared to nurses for both collecting swabs (median number of swabs per day: 46% (range, 0–96%) in physicians vs. 78% (range, 0–100%) in nurses, *P* = 0.042) and study diaries (median number of diaries per day: 47% (range, 0–97%) in physicians vs. 81% (range, 0–100%) in nurses, *P* = 0.033).

**Fig. 1:**
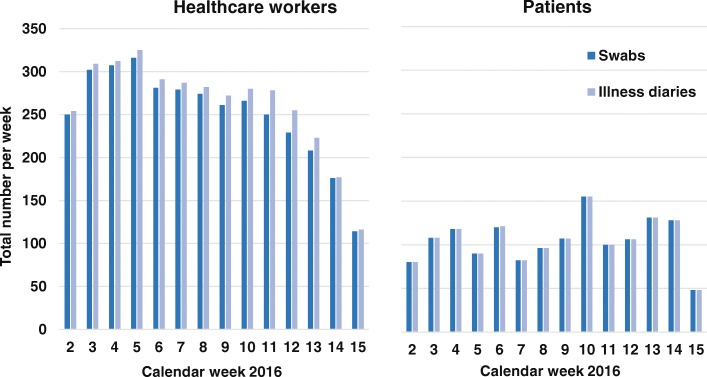
Number of swabs and illness diaries collected in healthcare workers and patients per calendar week, influenza season 2015/16

Figure 1 depicts the total number of swabs and diaries collected in HCWs (panel A) and inpatients (panel B) per calendar week. Given that the number of enrolled HCWs was stable over time, a marked decrease in the number of swabs and diaries could be detected after 9 weeks of study data collection, reflecting a decrease in protocol adherence. In hospitalized patients, however, such a decreasing trend in the number of samples was not observed.

## Discussion

In a prospective surveillance study of asymptomatic and symptomatic influenza infection conducted on three wards of a large university hospital during the influenza season 2015/2016, we were able to recruit 32% of inpatients and 62% of HCWs. HCWs provided self-collected swabs on 73% of all study days and illness diaries on 77%, whereas swabs and diaries could be obtained from inpatients in 83% of hospital days. Adherence to the study protocol after discharge was poor in hospitalized patients. Physicians provided a lesser proportion of swabs and diaries than nurses, and higher age, working in internal medicine and longer duration of participation were associated with better adherence among HCWs. Whereas the total self-collected swabs and diaries decreased over time in HCWs, adherence to the protocol was stable over time in hospitalized patients.

We were unable to locate any other study that followed cohorts of inpatients and HCWs on dedicated wards over one influenza season with the request to provide nasal swabs and diaries routinely irrespective of symptoms. Although our design is unique, we consider adherence to both illness diaries and swabs in more than 75% of study-days satisfying, which is supported by other authors who suggest to aim for response rates of at least 60% in surveys performed on a single point in time [20]. The high coverage in daily swabs and diaries highlights that, even considering decrease in adherence amongst HCW observed in later study periods, the majority of shedders must have been captured as influenza symptoms and viral shedding usually last more than a single day [21].

Although the problem of non-adherence in clinical trials is quite common for multiple reasons, [22] it can be improved by focusing on protocols and processes, even if they seem peripheral [23]. Frost et al. emphasize the importance of favorable trial conditions and organizational context, which can influence the quality of diary data collected [24]. We believe that daily presence of our study team on study wards together with a simple diary and high motivation of participating HCWs helped to achieve high adherence rates in our study despite its long duration. One other favorable factor may be the staffing conditions in our institution. It has a relatively high number of physicians and nurses per 100 hospitalizations compared to other institutions in our country, [25] and, as a university hospital, is experienced with clinical studies. The Swiss health system belongs to the five highest ranked countries with regard to personal health care access and quality, [26] which may also explain the high adherence rate, especially in an academic setting. Our HCWs may have more resources that allow them to participate in research activities. Barriers for adherence may have been the long study duration for HCWs with the need to remember swab collection and diary completion also during days off work and the efforts needed for patients to send the study materials back to the study office after discharge, in addition to differences in perceived importance of the need for full adherence to the protocol.

Our study has several strengths. In a well-defined study population within a single center, we were able to enroll and follow a substantial proportion of patients and HCWs over an entire influenza season. The critical proportion of participants, however, to detect influenza transmission events, has not been defined, and therefore a larger number of participants with higher adherence rates to the study protocol would certainly increase the probability of detecting influenza transmissions, especially from asymptomatic subjects. Nevertheless, incomplete data collection may still be adequate to address the question of detection of asymptomatic influenza transmission, as influenza virus shedding is expected to last for several days [27, 28]. The best adherence to the protocol was observed in the first half of the study period, and as influenza activity is usually high during the first four to 6 weeks of an influenza season, the decline in adherence to the study protocol towards the end of the study period is of lower significance (https://www.bag.admin.ch/bag/de/home/krankheiten/ausbrueche-epidemien-pandemien/aktuelle-ausbrueche-epidemien/saisonale-grippe---lagebericht-schweiz.html). Nevertheless, the risk of missing transmission events during the later stage of the season may be higher.

One limitation of our study is generalizability, as it was performed in specific wards and departments of a single university-affiliated institution in a high-income country. Our cohort of HCWs is relatively small and patients are diverse. It remains unclear whether the same recruitment rate and adherence can be observed in a different setting. We were not able to include all health care providers: our study population was limited to nurses, physicians and corporate hospitality staff working on the particular wards, and did not include other providers (e.g. physiotherapists, consulting physicians) who could also transmit influenza to study inpatients. In addition, we were unable to include visitors, who may also introduce influenza from the community.

## Conclusions

In conclusion, our study provides proofs of concept that it is possible to follow a cohort of both patients and healthcare workers in an adult acute care hospital setting and perform intensive surveillance for respiratory infections. The question whether this proportion of HCWs and inpatients is sufficient to reliably detect transmission events, especially from asymptomatic participants, remains open and deserves further study. Based on our results, other investigators should be encouraged to perform similar studies in order to investigate real-world clinical questions about the transmission dynamics of influenza infection.

## Abbreviation

HCW: Healthcare worker
